# Half-Yearly Compendium of Medical Science

**Published:** 1868-07

**Authors:** 


HALF-YEARLY COMPENDIUM OF MEDICAL SCIENCE—S. W. Butler, M. D. and D. G. Brinton, M. D., Editors. The first number of this work was issued in January, 1868. It has met with a cordial reception from the profession of this country, many of whom have given strong testimony to its value, both intrinsic and comparative. It fills a void in American Medical Literature, and aims to be *second to none of its class published.* One feature that emphatically recommends it to the medical profession everywhere, is the fact that while it contains a carefully prepared synopsis of foreign medical literature, that of our own country, which is annually growing in importance, is not neglected. None of the foreign abstracts do justice to American medical literature, being content with using the material found in scarcely half a dozen of our periodicals, thus practically ignoring by far the largest number, and many of the best of our medical writers.

The Compendium is published in January and July, containlng nearly 300 royal octavo pages, and is printed with good type on good paper, and is altogether gotten up in a *readable, attractive form.* Each department is paged independently, so that after a few years, title pages and indexes for each can be issued, thus giving the reader *separate volumes* on the several departments of medical literature. The consecutive paging of each number is at the bottom of the page.

We earnestly hope that this National undertaking will be heartily supported by the profession. Those wishing to subscribe, are reqnested to address Dr. S. W. BUTLER, 115 South Second street, Philadelphia, at once, *and not be backward about asking your neighbors to join you.*

The second number—for July—is in press, and will be ready about the middle of the month. B.

